# Trunk and upper arm postures and movements among older workers in physically demanding occupations

**DOI:** 10.1093/annweh/wxag027

**Published:** 2026-04-24

**Authors:** Charlotte Lewis, Farhad Abtahi, Mikael Forsman, Jens Wahlström, Andreas Tornevi, Pontus Öhrner, Viktoria Wahlström

**Affiliations:** Department of Epidemiology and Global Health, Umeå University, Umeå SE-901 87, Sweden; Division of Ergonomics, KTH Royal Institute of Technology, Stockholm SE-141 57, Sweden; Department of Clinical Physiology, Karolinska University Hospital, Stockholm SE-141 86, Sweden; Division of Ergonomics, KTH Royal Institute of Technology, Stockholm SE-141 57, Sweden; Institute of Environmental Medicine, Karolinska Institutet, Stockholm SE-171 77, Sweden; Department of Epidemiology and Global Health, Umeå University, Umeå SE-901 87, Sweden; Department of Epidemiology and Global Health, Umeå University, Umeå SE-901 87, Sweden; Sports Medicine, Department of Community Medicine and Rehabilitation, Umeå University, Umeå SE-901 87, Sweden; Department of Epidemiology and Global Health, Umeå University, Umeå SE-901 87, Sweden

**Keywords:** construction workers, kitchen workers, cleaners, assistant nurses, occupational physical activity, prolonged working life

## Abstract

**Introduction:**

Associations between work-related musculoskeletal disorders (WMSDs) and awkward postures and repetitive movements have been established. Action levels for elevated arms, trunk flexion, and fast movements have been suggested. With today's prolonged working life, older workers in manual jobs may be at increased risk for WMSDs. The aim of this study was therefore to assess postural workload in older workers in four physically demanding occupational groups using technical measurements and compare the exposure of the groups with each other's and with the action level.

**Methods:**

Postures and movements were measured for the right upper arm and the trunk using inertial measurement units (IMUs) during 3 workdays in construction workers (*n* = 35), kitchen workers (*n* = 37), cleaners (*n* = 27), and assistant nurses (*n* = 25), all over 50 years. Angle and angular velocity were computed, and time exceeding suggested angular cut-offs was assessed. Group mean and variance components within and between subjects were estimated in one-way random effect models. One-way ANOVA tests were conducted to compare exposure variables between the occupational groups.

**Results:**

Construction workers spent more time with arms >60° than other occupational groups (7.9% vs. 4.4–5.6%) and >90° (2.8% vs. 1.0–1.3%). Across groups, a substantial share of days exceeded suggested action level for arm elevation >30° (8.0–24.6%) and >60° (6.3–22.7%). Cleaners had the highest 50th percentile trunk flexion (15.3°), while construction workers spent the most time with trunk flexion >45° (12.6%) and >60° (7.4%); exceedances of recommended levels occurred in up to 39% of days. Kitchen workers and cleaners exhibited more time with fast arm movements (31.8–35.6% vs. 23.9–24.0%). Considerable variability was observed both within and between individuals.

**Conclusion:**

In the included occupational groups, in workers over 50 years, arm elevation and trunk flexion may often exceed recommended levels. Since the physical capacity is decreasing with age, this indicates elevated musculoskeletal risks, and there is a clear need for targeted ergonomic measures to support sustainable working lives.

What's important about this paper? Older adults in manual jobs may be at increased risk for work-related musculoskeletal disorders. This study described postures and movements of the right upper arm and trunk among older adults in four manual operations, and found considerable variability within and between individuals but that arm elevation and trunk flexion frequently exceeded recommended levels. The results indicate the need for preventive measures to enable sustained work among older adults, particularly given that physical capacity declines with age.

## Introduction

Musculoskeletal disorders (MSDs) constitute a large public health problem that causes not only personal suffering but also productivity loss and societal costs (GBD Disease and Injury Incidence and Prevalence Collaborators 2018 ; Safiri et al. 2020; Safiri et al. 2021). A substantial part of MSDs can be attributed to occupation, where in the UK, work-related MSDs accounted for 27% of all work-related ill health and 21% of all working days lost due to work-related ill health during 2022 to 2023, while in Sweden 65% of those with work-related problems report physical pain or ache related to work (Health and Safety Executive 2023; Swedish Work Environment Authority 2023). Work-related MSDs (WMSD) located in the neck, shoulders and lower back are common in occupational group with physically demanding jobs such as construction workers, cleaners, kitchen workers and healthcare workers (Jacquier-Bret and Gorce 2023, 2025; Greggi et al. 2024; Sánchez-Rodríguez et al. 2024; Santos et al. 2025) and there is a large body of occupational epidemiologic findings showing associations to awkward postures and repetitive movements of the back and shoulders (Farioli et al. 2014; Heneweer et al. 2011; Iqbal and Alghadir 2017; Jahn et al. 2023 ; Kuijer et al. 2018; Lewis et al. 2023; Linaker and Walker-Bone 2015; Mayer et al. 2012; Swedish Agency for Health Technology Assessment and Assessment of Social Services (SBU) 2022; van der Molen et al. 2017; van Rijn et al. 2010; Wærsted et al. 2020).

The prevalence of WMSD increases with age (European Agency for Safety and Health at Work 2020; Lewis et al. 2023), which can be a result of the ageing process. With age physical both aerobic capacity and muscle strength gradually declines, making older employees work closer to their maximum capacity and under a higher relative physical strain will be higher (Boss and Seegmiller 1981; de Zwart et al. 1995; Hamberg-van Reenen et al. 2009; Kenny et al. 2016; Westerståhl et al. 2025). Due to the ageing population, projections for European countries show a drastic change in the ratio of the number of people of working age to the number of retired (European Commission 2012; United Nations et al. 2019). This has led to successive increases in statutory retirement ages in many countries. However, 5% of the Swedish workforce aged 50 to 64 years claimed that their age is making them struggle to perform their work tasks and around 13% of the women and 8% of the men think that they will not be able to work until retirement (Swedish Work Environment Authority 2022).

In order to gain reliable data on postures, technical measurements are considered the superior method (Hansson et al. 2006 ; Teschke et al. 2009; Koch et al. 2016). A Swedish research group (Arvidsson et al. 2021) has suggested action levels for work with elevated arms as well as movement velocity. Suggested action levels are: arms above 30° more than 50% of the workday, arms above 60°, more than 10% of the workday, and 50th percentile movement velocity of the upper arms more than 60°/s. These levels have been suggested based on technical measurements derived from accelerometers (Arvidsson et al. 2021). For trunk postures and risk for low back pain, another Swedish research group (Lind et al. 2020) have, based on previous research, suggested a criterion of forward flexion above 45°, ≥1 h/d (12.5% of the workday) for high risk level.

There is a lack of studies assessing postural load in older workers with technical methods. Merkus et al. (2019) assessed postural load in construction workers and health care personnel over the age of 45 years, and found that the duration of arm elevation and trunk flexion >30° and >60° was similar between older and younger construction workers, but higher in older compared with younger healthcare workers. With only single studies looking into this, there is a need to increase knowledge about the physical demands of senior workers in physically demanding occupations. Therefore, this study aimed to assess the postural load in older workers across four physically demanding occupational groups, using technical measurements, and to compare them to suggested action levels. A secondary aim was to explore eventual differences between the occupational groups.

## Methods

### Study population

The current study is part of the HIPE project, which aimed to develop a technical method for assessing physical workload, to quantify cardiovascular workload and body postures among older employees in physically demanding occupations. Further the project aimed to investigate the factors that hinder or facilitate a prolonged working life (Wahlström et al. 2025a, 2025b). Workers (*n* = 129) in four different occupations (construction workers, kitchen workers, cleaners and assistant nurses) in northern Sweden were recruited in the study between August 2021 and April 2023. In the initial phase, organizational representatives, mainly human resources staff or line managers, were contacted to establish organizational commitment to the project and to obtain permission for employees to participate during working hours. Following this, employees received information about the study. The mode of communication varied across organizations and included different combinations of written materials, manager-mediated information, and oral presentations in which researchers introduced and explained the study during workplace meetings. The inclusion criteria were being ≥50 years old, employed at ≥50% of full-time equivalent, and working full-day work hours. Construction workers (*n* = 37, 0% women) were recruited from 12 organizations. Nineteen were carpenters (construction, assembly, and repair), eight painters (spackling and sanding, painting and wallpapering), seven floor layers (install, repair, and finish flooring materials), two concrete workers (prepare, pour, level, and finish concrete for construction projects) and one smith (shape, join, and repair metal objects). Kitchen workers (*n* = 37, 73% women) were recruited from seven organizations, including both public and private sectors, 27 were cooks, and 10 were kitchen assistants. Their main tasks were to carry out meal preparation and cooking, as well as supporting tasks such as cleaning and general kitchen maintenance. Cleaners (*n* = 29, 90% women) were recruited from five organizations, all of which were within publicly funded organizations, where the main tasks were maintaining cleanliness in office environments, including dusting, vacuuming, waste removal, sanitizing surfaces, and cleaning restrooms. Assistant nurses (*n* = 26, 92% women) were recruited from a regional healthcare organization in Västerbotten and two municipalities. Their work consisted of basic patient care in healthcare settings, including assisting with personal hygiene, mobility, feeding, and monitoring patients. Based on previous studies collecting similar exposure data we aimed to have at least 25 workers from each occupational group to have close to true mean values for the different groups (Wahlström et al. 2010, Wahlström et al. 2016). All subjects signed an informed consent form prior to entry into the study. The study was approved by the Swedish Ethical Review Authority (Dnr 2020-01927, 2020-05850, 2021-03400, 2023-01094-02).

### Data collection

All participants answered a questionnaire about their health, lifestyle, and work environment. General health was assessed with the question, “In general, you would say that your health is…” with five response alternatives between “excellent” and “poor” (Hays and Morales 2001; Orwelius et al. 2018). Leisure time exercise was assessed with the question, “How often have you worked out or exercised in training clothes during the last 3 mo, with the purpose of improving your physical capacity and/or your well-being?”, with five response alternatives from “never”, to “more than 3 times per week” (Ng et al. 2011). Work-postures were assessed with the questions “How much do you work with…” “forward bent posture” and “hands above shoulders”, with five response alternatives: “rarely”, “quite rarely”, “sometimes”, “quite often”, and “often”. These items have demonstrated evidence of predictive and concurrent criterion validity in relation to musculoskeletal symptoms and disorders in corresponding body regions (Engholm and Holmström 2005; Lewis et al. 2023; Wahlström et al. 2025a, 2025b).

### Technical measurements

For each worker, we assessed upper arm elevation and trunk forward sagittal inclination during three whole workdays. To improve readability, trunk forward sagittal inclination is hereafter termed trunk flexion. Inertial measurement units (IMU) (Movesense Oy, Vantaa, Finland) were used to measure postures and movements of the upper arms and trunk. The sensors were placed in an elastic T-shirt (Wergonic AB, Stockholm, Sweden), in customized pockets at the upper arms (just distally to the insertion of the deltoids) and upper back (at the level of T1–T2 vertebrae). The system has been tested for validity (Hoareau et al. 2023). The shape of the pocket and the extra sensor case with a matching shape were designed to prevent sensor rotation and limit relative movement errors. The shirt size, ranging from extra small to extra large, was chosen to be tight, but still comfortable for each participant. Both the accelerometer and gyroscope data from the IMU sensors were sampled at 26 Hz and transmitted to the Wergonic mobile application V1.9.4 via Bluetooth connections (Wergonic AB, Stockholm, Sweden). The system uses a sensor fusion algorithm to estimate body segment angles. If the angles were estimated based on gyroscope integration alone, drift would occur. In the fusion algorithm, accelerometer signals provide a gravity reference that continuously corrects low-frequency drift from the integrated gyroscope signals; therefore, the risk of drift in inclination angle estimates is minimized.

To obtain the coordinates of the zero angle of each upper arm, a zero-elevation reference position was recorded with the subject standing upright, leaning to one side with the arm hanging, relaxed, while holding a 2 kg dumbbell in their hand. For the trunk, a zero-trunk inclination reference position was recorded with the subjects standing upright (Lind et al. 2025).

### Data processing

Measuring days with less than 330 min of recorded data were excluded from the analysis. All data were visually inspected and days with erroneous data were excluded. To make the measured data and parameters on whole-day work postures and movement more comparable between different subjects and days, the first 6 h of measurement data were included in the analysis. Out of the 129 recruited participants one participant discontinued participation due to illness, and four participants did not have sufficient data to meet the inclusion criteria. The remaining 124 participants constituted the study population (Table 1). The system utilized zero-reference postures to compute angles for arm elevation (Weber et al. 2018) and forward bending, as well as generalized angular velocities for the arms and inclination velocity, ie the derivation of the inclination angle, for trunk movements, at a rate of thirteen samples per second (13 Hz) (Forsman et al. 2022a).

**Table 1:** Background characteristics of the study population (*n* = 124).

	All, *n* = 124	Construction workers, *n* = 35	Kitchen workers, *n* = 37	Cleaners, *n* = 27	Assistant nurses, *n* = 25
Age, mean (SD)	57.1 (4.2)	56.1 (3.8)	58.2 (4.4)	57.9 (4.2)	56.2 (3.9)
Women, *n* (%)	74 (59.7)	0 (0.0)	27 (73.0)	24 (88.9)	23 (92.0)
BMI, mean (SD)	26.9 (4.4)	26.2 (2.5)	26.8 (3.9)	28.1 (5.5)	26.9 (5.6)
Currents smokers, *n* (%)	6 (4.8)	2 (5.7)	2 (5.4)	0 (0.0)	2 (8.0)
Current snuff users, *n* (%)	23 (18.5)	11 (31.4)	5 (13.5)	4 (14.8)	3 (12.0)
General health, *n* (%)					
Poor	0 (0.0)	0 (0.0)	0 (0.0)	0 (0.0)	0 (0.0)
Fair	23 (18.5)	4 (11.4)	7 (18.9)	5 (18.5)	7 (28.0)
Good	66 (53.2)	22 (62.9)	20 (54.1)	13 (48.1)	11 (44.0)
Very good	33 (26.6)	8 (22.9)	10 (27.0)	9 (33.3)	6 (24.0)
Excellent	1 (0.8)	1 (2.9)	0 (0.0)	0 (0.0)	0 (0.0)
Leisure time exercise, *n* (%)					
Never	45 (36.3)	16 (45.7)	13 (35.1)	10 (37.0)	6 (24.0)
Not regularly	22 (17.7)	6 (17.1)	7 (18.9)	4 (14.8)	5 (20.0)
Once a week	17 (13.7)	2 (5.7)	3 (8.1)	6 (22.2)	6 (24.0)
2 to 3 times a week	31 (25.0)	9 (25.7)	13 (35.1)	5 (18.5)	4 (16.0)
>3 times a week	8 (6.5)	2 (5.7)	1 (2.7)	2 (7.4)	3 (12.0)

In contrast to inclination velocity, generalized velocity includes not only the inclination velocity, but the rotation around all three axes. The generalized velocity, which is about twice as high as the corresponding arm inclination velocity (Forsman et al. 2022a), has been used in many field studies (Lind et al. 2023). Down-sampled signals, with ten values per second (10 Hz), were saved to a text file (CSV), which was used for offline analysis. A simple report, including percentiles and time spent in different risk zones, was displayed immediately after the measurement, along with the corresponding suggested action levels (Arvidsson et al. 2021).

### Statistical analysis

The normality of all exposure variables was examined by the Shapiro–Wilk test. Since the variables, according to the tests, were normally distributed, mean values and standard deviations (SD) were presented in the results. Each individual's mean values of the 10th, 50th, and 90th percentiles of postures (upper arm elevation and trunk flexion, °) and movement velocities (°/s) across 3 d were calculated (Wahlström et al. 2016). The mean of the individuals’ values formed the group mean values of the four groups. Likewise, the proportion of time spent in neutral posture (<20°) and in postures exceeding 30°, 60°, and 90° angles was calculated, along with the frequency of periods lasting more than 3 s in neutral posture (Kazmierczak et al. 2005; Palm et al. 2018). The proportion of time spent at low velocities (<2.5°/s) and at high velocities (>45°/s) was also calculated. The velocity cut-offs (ie 2.5°/s and 45°/s) were set at half the values reported in previous studies, to enable comparison between our generalized velocity variables (derived from accelerometers combined with gyroscopes) and generalized velocity measures obtained from accelerometers alone. We also compared the 50th percentile velocity of our subjects to an action level converted from 60°/s to 30°/s, since going from using only accelerometers to using IMUs with accelerometers and gyroscopes has been shown to yield lower velocities (about 50% lower) (Forsman et al. 2022a, 2022b). One-way ANOVA tests were conducted to compare the assessed exposure variables between different occupational groups, using *P* < 0.05 to denote significant differences, with the exposure variables as dependent variables and occupational group as independent variable. For each exposure variable, variance components within and between subjects, for each occupational group, were estimated for the entire working day using one-way random effect models with restricted maximum likelihood algorithms. Exposure variability in each occupational group was expressed in terms of the SD between subjects (SD_BS_) and between days (SD_BD_) (Mathiassen et al. 2003; Mathiassen 2006).

## Results

The mean age of the participants was 57.1 (SD 4.2) years, and 59.7% of the participants were women. Eighty percent rated their general health as good, very good or excellent, 36% claimed to never engage in leisure time physical activity, and 32% exercised 2 times per week or more. For detailed background characteristics, see Table 1.

### Self-reported posture

Forty percent of the subjects rated that they worked with their arms above shoulder height often or quite often. This was most frequently reported in construction workers (74%) and least frequently reported in assistant nurses (8%). Working with bent or twisted back often or quite often was reported by 63%, ranging from 60% (assistant nurses) to 72% (construction workers).

### Postures and movements

**Figure 1:**
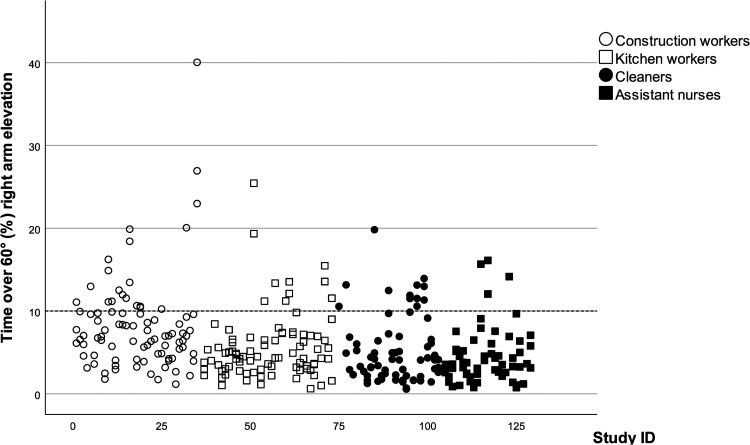
Time with right arm elevation >60° (%) for each day and participant, separated by occupational group. The dotted line represents the suggested action level.

There were, on average, 2.5 d of valid data per participant (a total of 308 valid measurement days), with an average duration of 359 min (SD 3.3) per day. For the right arm, the 50th percentile arm elevation angle was similar between the occupational groups (20.9° to 23.4°), while the 10th percentile was lower (6.2° vs. 7.7–9.0°) and the 90th percentile was higher (54.7° vs. 44.9–49.1°) in construction workers compared with the other occupational groups (Table 2). Construction workers spent a larger part of their workday with their arms elevated compared with other occupational groups (>60°: 7.9% vs. 4.4–5.6% and >90°: 2.8% vs. 1.0–1.3%). At the group level, none of the occupational groups exceeded the suggested action levels for work with elevated arms. There was however a large proportion of individual days where the workers exceeded the suggested action levels for work with arms >30° (8.0%, 15.9%, 24.6%, and 11.1%) and >60° (22.7%, 11.4%, 17.4%, and 6.3% of the days for construction workers, kitchen workers, cleaners and assistant nurses respectively) (Fig. 1).

**Table 2:** Postures (elevation) and movements for the right upper arm presented per occupational group.

		Construction workers (*n* = 35)	Kitchen workers (*n* = 37)	Cleaners (*n* = 27)	Assistant nurses (*n* = 25)	*P*
**Arm posture**						
10th percentile, °	Mean	6.2	9.0	7.7	7.9	0.03
	SD_BS_	2.7	3.7	4.0	3.1	
	SD_BD_	2.2	3.3	2.7	3.3	
50th percentile, °	Mean	21.9	23.4	22.3	20.9	0.43
	SD_BS_	5.1	4.7	6.4	4.7	
	SD_BD_	4.3	5.4	4.6	4.4	
90th percentile, °	Mean	54.7	49.1	46.1	44.9	0.00
	SD_BS_	12.3	6.7	9.4	7.4	
	SD_BD_	7.4	7.7	6.0	6.2	
Time in neutral (<20°), %	Mean	45.2	39.8	43.4	45.9	0.34
	SD_BS_	10.4	12.1	16.0	12.7	
	SD_BD_	10.0	13.5	11.9	12.2	
Time > 30°, %	Mean	31.7	33.4	31.5	28.6	0.54
	SD_BS_	9.8	11.0	13.7	9.4	
	SD_BD_	7.9	10.5	10.0	9.8	
Time > 45°, %	Mean	14.9	13.8	12.2	7.8	0.10
	SD_BS_	6.8	5.7	7.4	5.6	
	SD_BD_	4.8	6.1	4.6	4.0	
Time > 60°, %	Mean	7.9	5.6	5.0	4.4	0.00
	SD_BS_	4.7	2.4	3.1	2.7	
	SD_BD_	3.1	3.2	2.6	2.3	
Time > 90°, %	Mean	2.8	1.3	1.0	1.0	<0.001
	SD_BS_	2.8	0.8	0.4	0.6	
	SD_BD_	1.6	1.0	0.6	0.7	
Frequency of periods (>3 s) in a neutral posture/min	Mean	2.1	1.9	2.0	2.0	0.79
	SD_BS_	0.5	0.7	0.8	0.6	
	SD_BD_	0.5	0.7	0.7	0.5	
**Arm movement velocity**						
10th percentile, °/s	Mean	2.3	2.6	2.5	2.0	0.10
	SD_BS_	0.5	1.2	0.6	0.0	
	SD_BD_	0.7	0.8	0.9	0.8	
50th percentile, °/s	Mean	17.8	24.8	28.6	13.8	<0.001
	SD_BS_	5.6	7.6	9.8	2.7	
	SD_BD_	4.1	3.9	5.3	4.3	
90th percentile, °/s	Mean	77.2	94.4	101.0	85.4	<0.001
	SD_BS_	11.3	12.9	15.4	8.3	
	SD_BD_	7.5	5.6	6.4	8.8	
Time at low velocities (<2.5°/s, %	Mean	14.3	12.4	12.0	16.1	0.16
	SD_BS_	4.7	6.6	6.4	0.0	
	SD_BD_	7.1	6.5	6.7	10.4	
Time at high velocities (>45°/s), %	Mean	23.9	31.8	35.6	24.0	<0.001
	SD_BS_	5.5	6.3	7.9	2.5	
	SD_BD_	3.7	3.2	4.0	3.8	

The group mean values and standard deviations between subjects (SD_BS_) and between days within subject (SD_BD_) are presented for each group. The *P*-values display the difference in assessed exposure parameters between groups.

On average, kitchen workers and cleaners spent more time with fast movements of the right arm (31.8% and 35.6%, respectively) compared with construction workers and assistant nurses (23.9% and 24.0%, respectively). The average 50th percentile velocity for the right arm ranged between 13.8°/s and 28.6°/s, and if compared with converted action levels (30°/s) 21.1% of the days exceeded recommended 50th percentile velocity level (4.5%, 30.7%, 49.3%, and 0.0% of the days for construction workers, kitchen workers, cleaners and assistant nurses, respectively).

**Figure 2:**
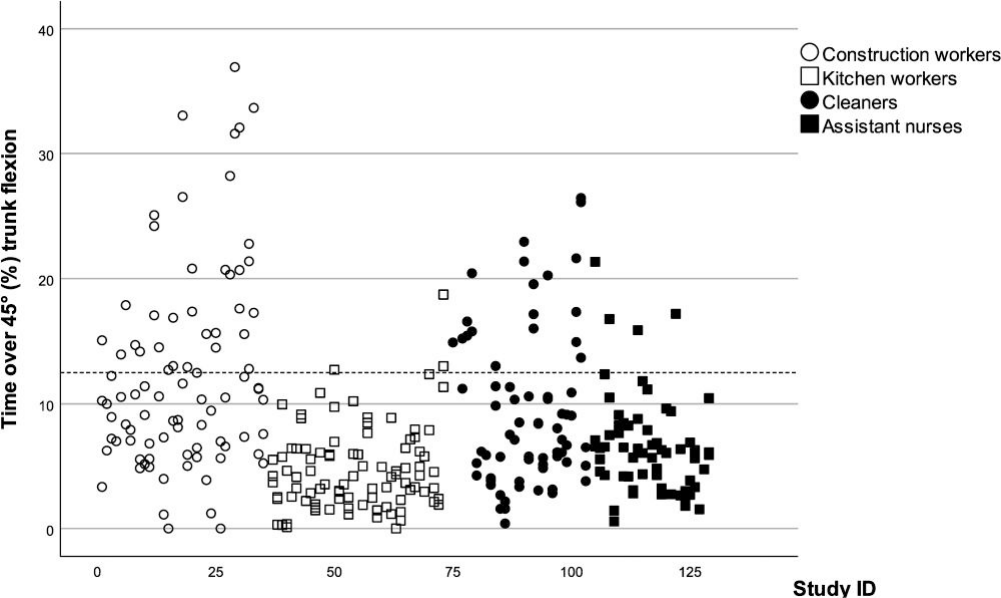
Time with trunk flexion >45° (%) for each day and participant, separated by occupational group. The dotted line represents the suggested action level.

The trunk flexion percentiles (10th and 90th) differed between the occupational groups, where construction workers had a larger range than the other occupational groups (Table 3). The 50th percentile trunk flexion for cleaners was 15.3° compared with 11.5°–13.1° in the other occupational groups. Time spent with >45° flexion was highest in construction workers with 12.6% of the workday, and 4.7%, 9.6%, and 6.6% in kitchen workers, cleaners and assistant nurses, respectively. Time with >60° trunk flexion was highest in construction workers (7.4%) and lowest in kitchen workers (2.2%). There were several individual days where workers exceeded the suggested action levels of time in trunk flexion >45° (39.1%, 3.3%, 27.5%, and 6.3% of construction workers, kitchen workers, cleaners and assistant nurses, respectively) (Fig. 2).

**Table 3:** Postures (flexion) and movements for the trunk presented per occupational group.

		Construction workers (*n* = 35)	Kitchen workers (*n* = 37)	Cleaners (*n* = 27)	Assistant nurses (*n* = 25)	*P*
**Trunk posture**						
10th percentile, °	Mean	−7.5	−3.6	−0.5	−1.3	0.001
	SD_BS_	5.9	6.7	4.0	5.4	
	SD_BD_	6.7	6.9	8.7	8.5	
50th percentile, °	Mean	12.5	11.5	15.3	13.1	0.081
	SD_BS_	4.7	4.4	4.5	4.2	
	SD_BD_	4.9	5.7	6.5	5.8	
90th percentile, °	Mean	48.2	32.2	43.5	36.9	<0.001
	SD_BS_	10.4	13.8	6.9	7.7	
	SD_BD_	9.5	8.9	11.5	10.3	
Time in neutral (<20°), %	Mean	64.0	70.4	59.5	64.5	0.011
	SD_BS_	9.7	10.3	8.8	10.1	
	SD_BD_	9.7	12.9	14.5	13.4	
Time > 30°, %	Mean	21.7	12.5	21.3	16.5	<0.001
	SD_BS_	6.8	9.1	4.9	7.1	
	SD_BD_	6.3	7.0	8.9	8.8	
Time > 45°, %	Mean	12.6	4.7	9.6	6.6	<0.001
	SD_BS_	6.2	2.5	5.7	2.1	
	SD_BD_	5.2	2.3	2.8	3.4	
Time > 60°, %	Mean	7.4	2.2	5.4	3.0	<0.001
	SD_BS_	3.2	4.9	1.6	2.3	
	SD_BD_	3.0	3.0	2.9	2.7	
Time > 90°, %	Mean	0.8	0.3	1.2	0.4	<0.001
	SD_BS_	0.5	0.8	0.5	1.1	
	SD_BD_	0.4	0.5	0.9	1.0	
Frequency of periods (>3 s) in a neutral posture/min	Mean	1.7	2.4	1.9	1.8	<0.001
	SD_BS_	0.4	0.4	0.2	0.0	
	SD_BD_	0.4	0.6	0.5	0.6	
**Trunk movement velocity**						
10th percentile, °/s	Mean	2.1	2.3	2.4	1.9	0.004
	SD_BS_	0.0	0.3	0.1	0.4	
	SD_BD_	0.8	0.7	0.7	0.9	
50th percentile, °/s	Mean	10.2	12.5	14.5	7.4	<0.001
	SD_BS_	2.9	4.2	3.1	3.5	
	SD_BD_	2.7	2.5	3.4	4.2	
90th percentile, °/s	Mean	46.7	56.0	53.5	46.1	<0.001
	SD_BS_	7.9	6.9	5.7	5.2	
	SD_BD_	5.2	5.3	5.6	5.9	
Time at low velocities (<2.5°/s), %	Mean	19.4	17.4	16.4	26.0	<0.001
	SD_BS_	5.4	3.2	5.9	5.9	
	SD_BD_	7.6	9.4	9.3	7.4	
Time at high velocities (>45°/s), %	Mean	10.9	14.7	14.3	10.4	0.001
	SD_BS_	6.2	2.5	5.7	2.1	
	SD_BD_	5.2	2.3	2.8	3.4	

The group mean values and standard deviations between subjects (SD_BS_) and between days within subject (SD_BD_) are presented for each group. The *P*-values display the difference in assessed exposure parameters between groups.

The 50th percentile trunk flexion velocity was highest among cleaners (14.5°/s) and kitchen workers (12.5°/s) and lowest in assistant nurses (7.4°/s) and a similar pattern was seen in time spent at high velocities (Table 3).

### Variability

We found considerable variability both between and within the workers (Figs. 1 and 2, Tables 2 and 3). For construction workers and cleaners, the between-worker variance was in general larger than the within-worker variance for the right arm, while there was less difference in the trunk variables. For the cleaners and the assistant nurses, the within-worker variance was larger than the between-subject variance for most trunk variables. The kitchen workers had a larger between-worker variance in the arm velocity variables.

## Discussion

Our study reveals that different occupational groups exhibit distinct postural exposures, with the construction workers showing greater daily total exposure time of both arm elevation and trunk flexion, cleaners demonstrating greater daily duration of trunk flexion, and kitchen workers and cleaners exhibiting greater daily duration of fast arm movements. A substantial number of days exceeded the time limits in awkward postures for both the arm and the trunk, especially among construction workers and cleaners. We also see variation between workers within each occupational group, as well as between days for the same workers.

### Comparisons with previous studies

The construction workers in the present study spent a comparable amount of time with arm elevation >60° as older construction workers (≥45 years) in a study by Merkus et al. (2019), but less than the mixed-age group of workers (18 to 65 years) reported by Palm et al. (2018). Regarding arm elevation >30°, our older workers exhibited shorter durations than those reported for older workers in Merkus et al. (2019), whereas time spent with arms >90° was similar to that observed in the construction workers studied by Palm et al. (2018). Trunk flexion time in our sample was slightly longer than that reported for the older construction workers in Merkus et al. (2019). It should be noted that Merkus et al. examined workers of similar age to those in the present study but collected posture data from a single workday, whereas Palm et al. based their results on four full workdays and included workers across all ages. Furthermore, neither study specified the type of construction workers involved or the work tasks performed.

Several studies on cleaners have presented similar levels of time with elevated arms and forward bending (Palm et al. 2018; Jørgensen et al. 2019). A Swedish study on fourteen female hotel cleaners presented substantially higher 50th percentile and 90th percentile arm elevation as well as arm velocity (Dahlqvist et al. 2018), even after converting the 50th percentile velocity to gyroscope measured (Forsman et al. 2022a), indicating that hotel room cleaning is even more physically demanding than the cleaning that was included in our study.

A study on older healthcare workers (≥45 years) presented time with upper arm >30° and >60°, similar to our results while working with trunk flexion was substantially higher than ours (Merkus et al. 2019). We have not found any studies on technically measured postures in kitchen workers to compare with our results.

None of the occupational groups had a mean exposure value that reached the proposed action level for time with elevated arms, and only construction workers did so for time in trunk flexion. However, all occupational groups spent several days with arm and trunk postures exceeding recommended levels. This is noteworthy in light of previous research demonstrating clear dose–response associations between time spent in unfavorable postures and the risk of long-term sickness absence. Gupta et al. (2022a) reported that as little as five additional minutes of forward bending >30° and >60° was associated with a 4% and 8% higher long-term sickness absence risk, respectively. Furthermore, Gupta et al. (2022b) demonstrated that even small increments in time spent with elevated arm postures were associated with an increased risk of long-term sickness absence. Against this background, our findings suggest that participants in the present study may be exposed to a substantially increased risk of long-term sickness absence. It should also be considered that our study population is, on average, older than those included in previous investigations, and could therefore exhibit greater susceptibility and therefore an even higher risk of disorders. This due to the decline in capacity (Westerståhl et al. 2025) which can result in working on a higher relative level compared with younger workers. However, we did not investigate differences in neither postural load nor physiological response between younger and older workers. Evidence on age-related differences in postural load is limited and inconsistent, with studies reporting no clear differences among construction workers, higher postural exposure among older healthcare workers, and lower trunk flexion among older metal workers (Merkus et al. 2019; de Zwart et al. 1995).

The velocities did not reach the suggested action level for any of the occupational groups on a group level, but for cleaners, half of the measured days exceeded the recommended 50th percentile velocity.

The relatively large between-subject and within-subject variance implies the necessity to measure on both several workers and days in order to get a valid group assessment for postures and velocities. The variance between subjects for time in extreme postures (>60°) was larger in construction workers compared with the other occupational groups. This can be explained by the diverse tasks and exposures within the construction trade. To accurately quantify work-posture-related exposures and identify associated risks, it is essential to measure these exposures based on specific occupations within the trade, for example, measuring specifically on floor layers, carpenters, and painters. This approach can provide higher resolution data, laying the groundwork for relevant and effective interventions to prevent WMSDs.

### Methodological considerations

This study included a relatively large number of technically measured participants and workdays in four different occupations with high physical demands. The occupational groups are homogenous in terms of being physically demanding and include only older workers (≥50 years), but they vary in terms of gender distributions as well as the private and public sectors. We measured for three consecutive days to capture variances between-days (within worker). However, we did not capture variations between weeks and months, which may occur in construction due to large project or phase variation. We also included lunch breaks in our measurements, which might lead to an underestimation of the times with postural load when compared with suggested action values, since they excluded lunch breaks. The action levels suggested by Arvidsson et al. (2021) are based on generalized velocity derived from accelerometers, whereas our velocity data is based on accelerometers combined with gyroscopes, making them not directly comparable. However, studies have suggested an approximate conversion between the two, which is why we compared 50th percentile velocity to 30°/s instead of 60°/s as is presented in Arvidsson et al. (2021) as well as defined “fast movements” as angular velocity above 45°/s instead of above 90°/s (Fan et al. 2021; Forsman et al. 2022a). This adjustment, however, renders them not directly comparable. There are studies in which inclination angular velocity has been used for the arms. Inclination velocity is lower than generalized velocity, which needs to be accounted for when comparing different studies (Forsman et al. 2022a). Further, we were not able to differentiate whether the arm movements were supported or unsupported, nor capture any external load during work, which will influence the physical load on the worker (Feng et al. 1997). We did not perform a post-recording reference posture to assess potential sensor displacement, as may occur during prolonged or highly dynamic tasks. Embedding IMUs in tight-fitting garments as what was used in the present study has been found to reduce this risk (Lind et al. 2025).

Construction and kitchen workers were recruited from a range of trades and sectors, providing broad occupational coverage. In contrast, cleaners and assistant nurses were recruited from publicly funded and labor-intensive settings only; thus, exposure estimates for these groups should not be generalized to all cleaners or assistant nurses, as exposures may differ in other work contexts.

## Conclusion

Among workers over 50 years of age, arm elevation and trunk flexion frequently exceeded suggested action levels, particularly among construction workers and cleaners. Moreover, kitchen workers and cleaners exhibited higher movement velocities compared with construction workers and assistant nurses. These exposure patterns suggest that older employees may face elevated musculoskeletal risks, underscoring the need for targeted ergonomic interventions to support sustainable working lives.

## Data Availability

The data that support the findings of this study are not openly available due to reasons of sensitivity and are available from the corresponding author only upon reasonable request. Data are located in controlled access data storage at Umeå University.
